# Tearing Aside the Veil

**Published:** 1907-03

**Authors:** 


### Tearing Aside the Veil

A SUIT in equity has been brought in the superior court at Concord, N. H., for the purpose of securing an accounting of the receipts and expenditures of the fortune of Mrs. Mary Baker G. Eddy, pastor emeritus of the Christian Science Church and author of all the works on christian science which amount to anything in a financial sense. The appointment of a receiver is also asked. The plaintiffs are Mrs. Eddy's only son, George W. Glover, his daughter, Mary Baker Glover, and Mrs. Eddy's nephew, George W. Baker. The real petitioner is Mrs. Eddy herself. The New York World which brought to public attention a fact suspected by many outsiders and generally known within the inner circles of the church, that Mrs. Eddy was mentally and physically incapacitated and was being kept in seclusion while a living dummy was sent out in her stead on the daily drives, published on March 2 the facts connected with the bringing of the suit.

It is interesting reading and in view of the fact that the defendants named are the members of the Eddy household at Pleasant View, it is quite likely that revelations of a more or less intimate character will be forthcoming. The papers tell a most pathetic story of the inability of the son to reach his mother without interference from the servants of the house. His letters were read by others and answered by them; and when he finally did gain access to the presence of his mother, a feeble and tottering old woman greeted him and his daughter, an old woman rapidly approaching her grave, who babbled of a "stolen will," and a plot to kill her; whose mind wavered and flickered between the events of today and the happenings of tomorrow, and to whose memory, age-crumbled and fleeting, the things of a week ago were lost in the musty ages of a past long, long forgotten.

This suit is not an attack on the Christian Science Church or any of its beliefs; they do not enter into the case at all; the action is for the protection of an old woman who is under the influence of people surrounding her who are not laboring in her interests. Vast sums of money are involved and the revelations which are to come when the trial is reached will be amazing in their ramifications and world-wide extent. That there is nothing in the nature of a circus display or tom-tom hurrah about the action, is shown by the fact that the senior counsel for the plaintiffs is ex-senator William E. Chandler, of New Hampshire.

It is more than likely that there will be considerable science of a certain financial character disclosed in the trial; but just how much christianity will be shown remains to be seen.

---

THE medical inspection of schools is an important question for the municipality to deal with. A large gathering of prominent women met the aldermanic ordinance committee recently, at which several of them spoke in an intelligent and telling fashion. The health commissioner, Dr. Wende, has included in his estimates for the coming year an item of $2,500 with which to employ two physicians at $1,000 each and one nurse at $500, to provide for such inspection. This is altogether too small an amount and the inspectors are too few, but it would make a substantial beginning and is perhaps adequate to test the practicability of the plan. We submit, however, that the only way to inspect is TO INSPECT!! Let the Common Council provide the ways and means at once in order that the important work can begin without delay.

---

DR. E. H. PORTER, the state commissioner of health, recently issued the twentieth annual report of his department, which is probably the most comprehensive survey of the questions belonging to the public health problem in this state that has been made in recent years. It is our purpose at this time, because of limited space, to refer to but one topic—namely, the registration of vital statistics. There is room for decided improvement in this branch of the public health work. The commissioner recommends a bill providing for the filing of all birth, marriage and death certificates outside of Greater New York in the state department. At present Buffalo, Albany and Yonkers file their own. The state should have a complete file of these records. The legislature should make haste to provide adequate means to improve the registration of vital statistics on the lines recommended by the commissioner.

---

LIEUTENANT JAMES CARROLL of the Army Medical Corps rose from the ranks to his present position. He rendered valuable service as a member of the army board for the study of infectious diseases in Cuba and was associated with Major Walter Reed in his studies and experiments with the yellow fever mosquito. Dr. Carroll submitted to the bite of an infected mosquito and suffered a severe attack of yellow fever as the result. It is proposed to promote him to major in the army by special act of congress as a reward for his invaluable services. It should be done without hesitation.

